# Do Gut Microbiomes Shift After Bariatric Surgery? A Literature Review

**DOI:** 10.3390/medicina61050849

**Published:** 2025-05-05

**Authors:** Zofia Sorysz, Piotr Kowalewski, Maciej Walędziak, Anna Różańska-Walędziak

**Affiliations:** 1 Medical Faculty, Collegium Medicum, Cardinal Stefan Wyszyński University in Warsaw, 01-938 Warsaw, Poland; zofia.sorysz@student.uksw.edu.pl; 2 Department of General Surgery, Military Institute of Medicine—National Research Institute, Zegrzyńska 8, 05-119 Legionowo, Poland; 3 Department of General, Oncological, Metabolic and Thoracic Surgery, Military Institute of Medicine—National Research Institute, Szaserów 128 St., 04-141 Warsaw, Poland; maciej.waledziak@gmail.com; 4 Department of Human Physiology and Pathophysiology, Faculty of Medicine, Collegium Medicum, Cardinal Stefan Wyszynski Universityin Warsaw, 01-938 Warsaw, Poland; aniaroza@tlen.pl

**Keywords:** gut microbiota, Firmicutes, Bacteroidetes

## Abstract

The human gastrointestinal tract is estimated to be populated by 38 trillion bacteria from almost 1000 different species. The dominant phyla are Firmicutes, Bacteroidetes, Actinobacteria, and Proteobacteria. However, the diversity and amount of gut microbiota depends on various factors. The importance of gut microbiota is increasingly noticed due to the influence of bacteria on energy homeostasis, the immune system, general health, and metabolism. Bariatric surgery is the mainstay treatment for patients with obesity. Two of the most common mechanisms are reducing gastric volume and decreasing ghrelin secretion. This literature review aims to depict the diverse impact of different bariatric procedures on gut microbiota. The original research papers were collected from the PubMed, Cochrane, and Elsevier databases. This literature review is focused on human studies. However, several references include animal models, specifically rats and germ-free mice. The findings suggest that bariatric surgery causes changes in the diversity of gut microbiota. However, the specificity of the changes depends on the type of bariatric surgery. The Firmicutes/Bacteroidetes ratio is elevated in the groups of patients with obesity compared to lean individuals. Bariatric surgery lowers the ratios impact on metabolism and energy absorption. Gut microbiota produces short-chain fatty acids, of which butyrate is responsible for strengthening the gut barrier, and acetate is correlated with fat deposition and lipogenesis. Moreover, changes in short-chain fatty acids influence insulin resistance and inflammation. In conclusion, bariatric surgery impacts gut microbiota, resulting in metabolic changes in patients, and the need for further study regarding long-term microbiota alterations post-operation is notable.

## 1. Introduction

Gut microbiota (GM), also called the “second brain”, describes the 38 trillion microorganisms living in the human gastrointestinal tract [1]. The microbiota exists in a mutualistic relationship with our body. The metabolites produced by microorganisms impose several effects on the organism of the host or other bacteria populations, such as metabolism regulation, the absorption of nutrients, and GM composition regulation [2]. Vitamin B12 is known to be produced by *Cetobacterium somerae* and was found to have an effect on tightening the junctions in the gut barrier to prevent pathogen infections [3]. Additionally, GM has a significant role in regulating systemic responses to different pathogens [4]. The term “second brain” describes the microbiota–gut–brain axis, established by the neuromodulatory metabolites produced by microbiota, such as the precursors of tryptophan and serotonin [5].

Because the stomach’s acidity reduces the ability of bacteria for adaptation, gastric microbiota is less diverse, as the low pH of gastric fluid allows only for acid-resistant bacteria such as *Helicobacter pylori* to survive. *H. pylori* is involved in the pathogenesis of gastric ulcer disease and, over time, may increase the risk of oncogenesis. Other bacterial genera found in the gastric cavity include *Streptococcus* spp. and *Prevotella* spp. The following parts of the gastrointestinal tract—duodenum, small intestine, and large intestine—are inhabited predominantly by the phyla Firmicutes, Bacteroidetes, Actinobacteria, and Proteobacteria [6,7]. Based on recent research regarding microbiome shifts in ICU patients, we can observe a significant implication of microbiome shifts and their influence on morbidity and mortality on the patients. Moreover, the influence of microbiome on morbidity and mortality after bariatric surgery is yet to be studied.

Due to the established link between gut microbiota dysbiosis and obesity, it is crucial to understand how bariatric surgery, as the treatment for obesity, may further influence GM.

The purpose of this review was to analyze the possible changes in the gut microbiome of patients who underwent bariatric surgery. Bariatric surgery is the mainstay of treatments for patients with obesity. The most important mechanisms for the reduction of excess body weight are the reduction of gastric volume and a decrease in ghrelin secretion, leading to reduced nutrient consumption and absorption. Bariatric surgery has a high safety level, with low incidence of perioperative complications and a low mortality rate of 0.04–0.3% [8]. The influence on gut microbiota may differ depending on the type of surgery, as some operations that include intestine interposition, such as Roux-en-Y gastric bypass (RYGB) and biliopancreatic diversion with duodenal switch (BPD/DS) affect the gut microbiome in a more distinctive way.

## 2. Materials and Methods

**Figure 1:**
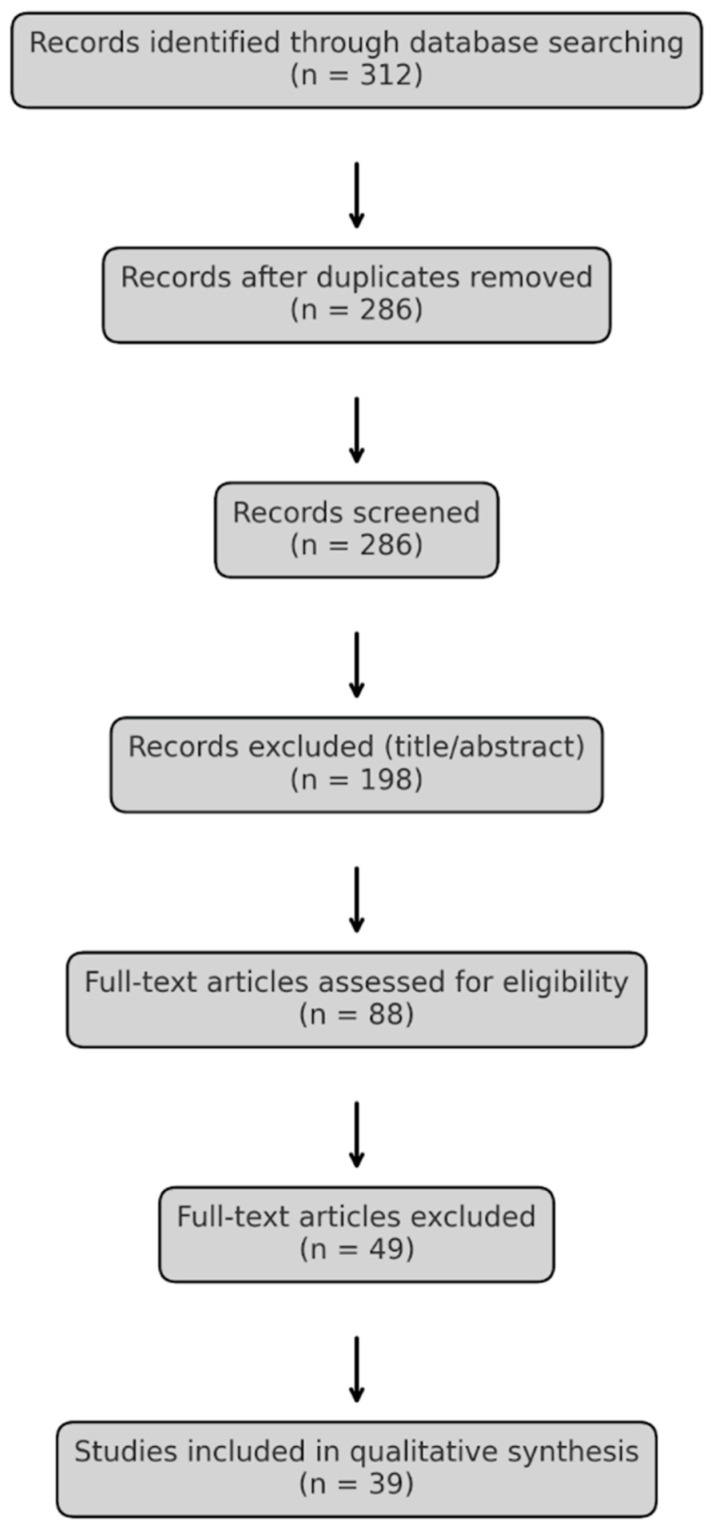
PRISMA flowchart showing the selection of studies included in the narrative review on gut microbiota alterations following bariatric surgery.

This review article is based on original research conducted in January 2025 of the National Library of Medicine, PubMed, Elsevier, and Cochrane databases, which were comprehensively searched for the phrases “gut microbiota”, “gastric microbiota”, “gastrointestinal tract microbiota”, and “bariatric surgery”, not including the word “review.” The search was limited to English-language studies published between January 2012 and January 2025. The results are based on exclusively original research and systematic reviews. This literature review is focused on human studies. However, several references include animal models, specifically rats and germ-free mice. The inclusion of specific studies was based on methodological rigor, relevance to the topic, and clarity in reporting microbial shifts in relation to bariatric surgery outcomes. The included studies met the following criteria: human or animal model studies focused on microbiota composition before and after bariatric surgery, and studies with reported microbial taxonomic changes at phylum, genus, or species level. The articles that were assessed for quality and relevance were the ones that met most, but not all, of the inclusion criteria before being excluded or included on a case-by-case basis. The PRISMA chart showing the inclusion of studies is presented below (Figure 1). The manuscript is divided into sections based on the type of bariatric procedure (Roux-en-Y gastric bypass, sleeve gastrectomy, mini-gastric bypass) with the purpose of analyzing whether there are changes in the gut microbiota in post-bariatric patients.

## 3. Results

**Figure 2:**
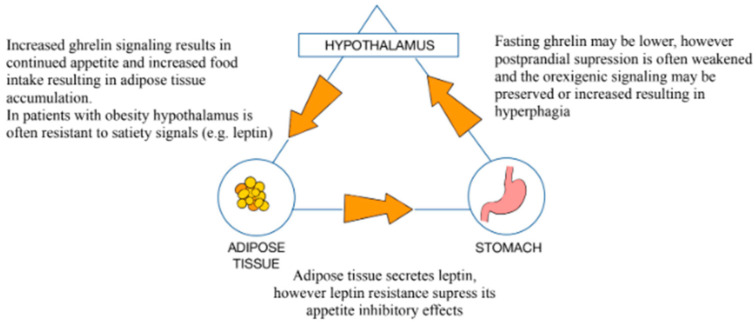
Ghrelin secretion and its regulatory pathways in patients with obesity: stomach–hypothalamus–adipose tissue axis.

**Figure 3:**
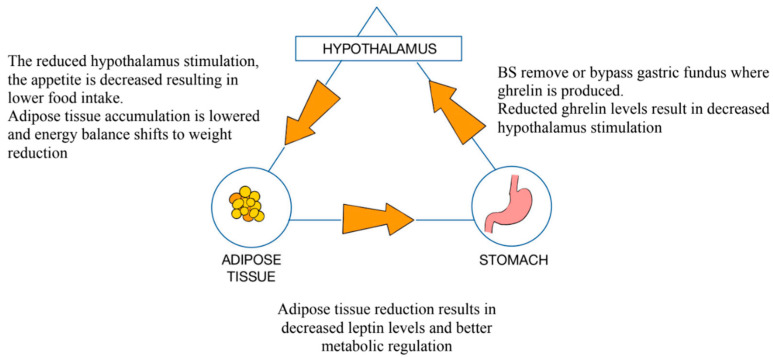
Ghrelin secretion and its regulatory pathways in patients post-bariatric surgery: stomach–hypothalamus–adipose tissue axis.

**Figure 4:**
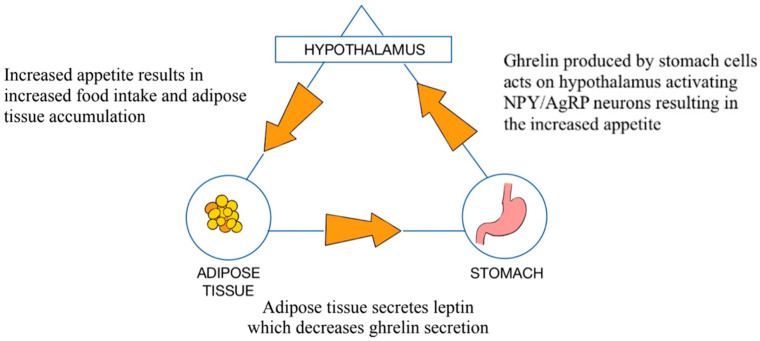
Ghrelin secretion and its regulatory pathways: stomach–hypothalamus–adipose tissue axis.

Obesity has become one of the most important healthcare issues in high-income countries and is linked with type 2 diabetes mellitus (DM2), cardiovascular disease, and a higher risk of oncogenesis. The World Health Organization (WHO) defines obesity as a Body Mass Index (BMI) exceeding 30 kg/m^2^. Generally, obesity is correlated with decreased GM diversity [9]. A study by Palmas et al. presented that there is an abundance of Bacteroidetes (classes: Flavobacteriia, Bacteroidia, and Sphingobacteria) and an increased population of Firmicutes (classes: Clostridia, Bacilli, and Negativicutes) found in patients with obesity when compared to the general population [10]. Obesity is linked to significant microbial dysbiosis, which is negatively correlated with patients’ BMIs [11]. Obesity is also associated with an increased count of Enterobacteriaceae (e.g., *Escherichia coli*), producing excess lipopolysaccharides (LPSs) [12]. Meanwhile, probiotic bacteria such as *Bifidobacterium* spp. and *Lactobacillus* spp. are often less abundant, which leads to a deterioration of the gut barrier and the promotion of inflammation [13,14]. (Figure 2, Figure 3, Figure 4)

### 3.1. Role of the GM in the Pathophysiology of Obesity

Obesity is triggered by an imbalance between energy intake and expenditure, as a result of genetic susceptibility and environmental, nutritional, social, and physiological factors [15,16,17]. The influence of gut microbiota on the metabolism and energy homeostasis of an organism is becoming increasingly recognized. Anaerobic fermentation performed by GM is responsible for altering the absorption of nutrients by reducing undigested polysaccharides, such as fiber, into short-chain fatty acids (SCFA), such as butyrate, acetate, and propionate, which influences lipid and glucose metabolisms [18]. The glucose metabolism is altered by SCFAs activating the free fatty acid receptor 3 (FFAR3) and stimulating the secretion of peptide YY (PYY), released by the endocrine system. PYY acts through the hypothalamus, leading to a reduced appetite [19,20]. Moreover, SCFAs provide the substrate for lipid synthesis [21] and are crucial for maintaining and regulating gut integrity, pH, and mucus production [22]. The high value of the Firmicutes/Bacteroidetes ratio in patients with obesity caused by Firmicutes overgrowth enhances fiber fermentation and contributes to excess SCFA production, creating an additional energy source. This results in provoking increased caloric absorption and the deposition of fat tissue [23]. The imbalance in the SCFA proportion, with higher levels of acetate, results in elevated lipogenesis and the deposition of fat in the liver and fat tissue, while decreased butyrate levels, responsible for the proper functioning of the gut–blood barrier, lead to inflammation and insulin resistance [24].

### 3.2. Intestinal Hyperpermeability

The damage to the structure of the intestinal barrier caused by gut dysbiosis is a condition commonly called “leaky gut”. A deterioration of the gut–blood barrier enhances permeability, enabling microorganisms, SCFAs, and bacterial toxins such as LPS to dissolve into the bloodstream and activate toll-like receptors 4 (TLR4) on the immunity cells, leading to chronic inflammation and insulin resistance. This state is linked to the development of DM2, depicted by chronic low-grade inflammation and insulin resistance [25,26]. Studies performed in germ-free mice with *Escherichia coli* overgrowth showed elevated levels of LPS in the serum, an increased penetration of the adipose tissue by macrophages, and higher cytokine secretion [27]. Overpopulations of Enterobacteriaceae and *Escherichia coli* are associated with obesity and a high-fat diet, resulting in elevated LPS levels in human serum. Bariatric surgery can influence gut permeability by reducing gut dysbiosis through changes in the composition of gut microbiota and the expression of tight junction (TJ) proteins. The depletion of LPS-producing bacteria and improvement of the F/B ratio results in a reduction of inflammation.

### 3.3. The Firmicutes/Bacteroidetes (F/B) Ratio

Firmicutes and Bacteroidetes are the main components of the human gut microbiota, comprising more than 90% of the bacterial population. The Firmicutes/Bacteroidetes (F/B) ratio is significantly higher in patients with obesity compared to normal-weight individuals. The relative abundance of Bacteroidetes may be reduced by as much as 90%, while the proportion of Firmicutes increases in individuals with obesity compared to those with a BMI lower than 25 kg/m^2^ [28,29].

The changes in F/B ratio are recognized as a hallmark for obesity prediction and other pathological conditions affecting the gastrointestinal tract, such as the diarrhea variant of irritable bowel syndrome (IBS-D). Additionally, the possible consequences of F/B ratio changes include the areas of SCFA metabolism, inflammation, and insulin resistance. The higher F/B ratio in patients with obesity may contribute to a greater capability of energy extraction from the food consumed. In the literature, some controversy regarding the F/B ratio exists due to population-specific factors and diet. There were many studies conducted in cohorts of animals with obesity that showed changes in the F/B ratio, but the clinical utility of the F/B ratio in humans is yet to be established [30]. Recent studies on F/B ratio between normal-weight subjects and subjects with obesity show limitations, which may be due to co-variabilities affecting microbiota composition [31]. Ethnicity, geography, lifestyle, and baseline diet affect microbiota composition. High-fiber, polyphenol-rich diets promote the stability of a microbiome; meanwhile, processed food, as well as high-fat and high-sugars diets can disrupt microbiota composition and affect the F/B ratio. Along with Firmicutes and Bacteroidetes, the human gut microbiome also comprises other phyla, including Proteobacteria, Actinobacteria, and Verrucomicrobia [32].

### 3.4. Gut Dysbiosis

Gut dysbiosis is defined as a dysregulation in the composition and function of GM. In patients with obesity, the diversity of GM is often decreased, and the population of pathogenic species outweighs that of beneficial species. The most common shifts in GM associated with obesity include an increased abundance of Firmicutes (e.g., *Clostridium* spp.), *Prevotella* spp., and *Methanobrevibacter* spp., accompanied by a deficiency in beneficial genera such as *Bacteroides* spp., *Bifidobacterium* spp., *Lactobacillus* spp., and *Akkermansia* spp. [33]. *Akkermansia* spp. perform a role in appetite and metabolism by increasing GLP-1 secretion, reducing metabolic inflammation. Additionally, there is a significant role of *Akkermansia* spp. in enhancing the stability and function of gut–blood barrier. Reduced ghrelin and improved glucose metabolism are associated with *Lactobacillus* spp. The deficiency in those significant species and the overpopulation of the detrimental species result in gut dysbiosis. The dysbiotic GM can cause inflammation by activating TLRs and increasing inflammatory cytokines, including IL-6 and TNF-α, which damage the gut epithelium. This results in chronic inflammation that can lead to autoimmune diseases, for example diabetes mellitus [34]. Furthermore, gut dysbiosis can contribute to deteriorated myeloid cell development in bone marrow [35]. The maturing of the innate immune system, neutrophils and DC, is disturbed, leading to reduced interferon type I (INF-1) and IL-15 secretion and a decreased capacity to kill the pathogens. This may lead to a higher risk of developing IBS and atopic diseases, including asthma and atopic dermatitis in adulthood [35,36,37]. The therapeutic strategies for reversing GM dysbiosis include dietary changes, probiotic and prebiotic supplementation, and fecal microbiota transplant (FMT). FMT is also considered to be effective in treating a recurrent Clostridioides difficile infection (CDI).

### 3.5. Roux-en-Y Gastric Bypass (RYGB)

RYGB is a bariatric procedure that aims to minimize gastric volume and the intestinal absorption of nutrients. A stomach-like pouch is created, with a significant reduction of gastric volume, and bypassing the duodenum reduces the absorption of nutrients. In a study conducted by Li et al., GM were compared between patients who had undergone RYGB, a sleeve gastrectomy (SG), or laparoscopic gastric banding (LGB). The patients who underwent RYGB showed the most significant change in their GM [38,39]. Microbiota alteration after RYGB may be due to pH fluctuations and changes in the gut environment. A higher pH is associated with microbial overgrowth, and increased levels of *Veillonella* spp., *Acidaminococcus* spp. (class Negativicutes), *Slackia* spp. (class Actinobacteria), *Granulicatella* spp. (class Bacilli), *Akkermansia* spp. (class Verrucomicrobiae), *Escherichia* spp., and *Klebsiella* spp. (class Gammaproteobacteria) were observed [40,41].

Tremaroli et al. analyzed the GM of a group of nine women with obesity who had not undergone bariatric surgery and compared them with a group of seven women who had undergone RYGB. The results showed an increase in Gammaproteobacteria and a significant decrease in *Gemella* spp. (class Bacilli), *Clostridium difficile*, and *Clostridium hiranonis* [42].

After RYGB, patients are observed to have a bacteria population more capable of producing tyramine from tyrosine and converting phenylalanine to phenylacetate and tryptophan to indole and tryptamine than non-surgery individuals without obesity [38]. A study by Shi et al. showed a difference between fecal samples taken from patients before, 1 month, 3 months, and 6 months post-RYGB. A considerable decrease was observed in the population of the families Lachnospiraceae and Ruminococcaceae [43]. Lachnospiraceae are responsible for butyrate production, SCFAs originating from the fermentation of fiber. By strengthening the gut–blood barrier, butyrate helps to isolate toxins and pathogens from blood and reduces inflammation [44]. However, the correlation between the decrease in the Lachnospiraceae population and lowered permeability is yet to be studied. A study by Casselbrant et al. on the gut–blood barrier and TJ protein alternations after RYGB shows that there is a significant increase in claudins-3 and claudins-4 expressions and a decrease in ZO-1 and occludin expressions observed 6–8 months post-operation [45]. The reduction of the production of butyrate by occludin, ZO-1, and Lachnospiraceae may sensitize the gut barrier, resulting in “leaky gut” syndrome, a condition in which toxins, bacterial fragments (e.g., LPS), or undigested food components can penetrate through the intestine to the bloodstream. The increased permeability of the blood–gut barrier for LPSs may trigger compensatory mechanisms, such as the upregulation of claudins-3 and claudins-4, in an attempt to restore the integrity of the barrier. LPSs perform an important role in an immune response by activating CD14/TLR4 receptors and provoking an immune response. This pro-inflammatory response can commonly be observed in patients with obesity and results in consequences such as diabetes mellitus type 2. The LPS concentrations in the group of patients post-BS are observed to be lower than of pre-surgical patients [46].

Additionally, there is an increase in microbiota metabolite concentrations, such as Trimethylamine-N-oxide (TMAO), which is used to predict cardiovascular risk and arteriosclerosis [36]. TMAO is a biologically active metabolite eliminated mainly by renal excretion. Current studies suggest that TMAO blood concentration is positively correlated with endothelial dysfunction, inflammation, and the activation of thrombocytes [40]. The inflammation process is triggered by TMAO through an increased production of pro-inflammatory cytokines, such as interleukin 1-beta (IL-1β), tumor necrosis factor-alpha (TNF-α), and IL-6 [47]. A study by Trøseid et al. showed that TMAO blood concentration more than doubled 1 year post-operation, compared to the pre-operative TMAO blood concentration in patients with obesity prior to lifestyle changes (a 10% weight loss was required for the patients to have qualified for bariatric surgery). The results were innovative as TMAO had been previously associated with a higher risk of cardiovascular disease (CVD). Meanwhile, bariatric surgery is known to reduce the risk of CVD due to body weight loss and a remission of co-morbidities [48]. Therefore, the correlation between atherosclerotic cardiovascular disease (ASCVD) and TMAO concentrations needs further investigation. GM shift is presented in Table 1.

### 3.6. Sleeve Gastrectomy (SG)

An SG aims to reduce stomach volume by 85% while simultaneously minimizing the amount of food consumed by patients to approximately 150 mL. The low rate of perioperative complications, the technical feasibility and short duration of the procedure are reasons for an SG to be the preferable bariatric surgery in many countries, including the USA [49]. Murphy et al. described an increase in the population of Bacteroidetes spp. after an SG [50]. Other studies analyzing the GM changes after an SG showed an increase in Bacteroidetes and Actinobacteria populations, as well as *Blautia* spp., *Akkermansia* spp., *Eubacterium* spp., *Lactobacillus* spp., *Cyanobacteria* spp., and *Haemophilus* spp. However, an abundance of the Firmicutes population was observed [51]. An increased Bacteroidetes count, a decreased Firmicutes count, and a lower F/B ratio was present in patients 12 months post-SG [52]. A higher Bacteroidetes population enhances FXR/TGR5 signaling, resulting in an improvement in glucose metabolism, lipid homeostasis, and energy balance. A study by Yung et al. about microbial function and metabolic pathway alterations after an SG and RYGB in rats presented an improved ketone body synthesis ability and degradation due to GM alternations after an SG. Microbiota’s capability of glycerophospholipid metabolism and sphingolipid metabolism is enhanced [53]. Cao et al. observed an effect of an SG on decreasing pro-inflammatory cytokine concentrations such as IL-6, IL-1β, IL-18, and IL-23 post-surgery. An SG alters gut microbiota by decreasing *Desulfovibrio* spp. levels, a pro-inflammatory microbe, and increasing *Lactobacillus* spp. levels., an anti-inflammatory microbe [54]. Additionally, an overgrowth of anti-inflammatory species populations, such as *Lactobacillus* spp. or *Akkermansia muciniphila*, can limit TMAO production. Microbiota-derived metabolites such as SCFAs activate G-protein-coupled receptors in intestinal and adipose tissues, enhancing GLP-1 and PYY secretion [55]. As a result, an improvement in insulin sensitivity is observed. Moreover, SCFAs decrease hepatic gluconeogenesis and increase glycogen synthesis [56]. Butyrate stabilizes the gut–blood barrier and reduces metabolic inflammation [57].

### 3.7. Mini-Gastric Bypass (MGB)

Mini-Gastric Bypass, also known as a single-anastomosis gastric bypass (SAGB) or one-anastomosis gastric bypass (OAGB), was developed as a simplification of the RYGB procedure. This technique includes creating only one anastomosis, which shortens the time of the surgery and lowers the technical difficulty, with a reduction of further complications and faster patient recovery [58]. The International Federation for the Surgery of Obesity and Metabolic Disorders (IFSO) started to implement the surgery nomenclature as the mini gastric bypass-one anastomosis gastric bypass (MGB-OAGB) [59]. MGB helps with weight reduction, with comparable outcomes and risk profiles to RYGB; concurrently, it improves glycemic control and lipid profiles [60]. Compared to RYGB, the alternation in the abundance of beneficial GM species in a group of patients undergoing MGB is yet to be studied, as there is a notable limitation of research regarding MGB available [61].

### 3.8. Metabolic Improvements and Individual Variability in Microbial Shifts After Bariatric Surgery

The microbiome responses to bariatric surgery are highly individual and may differ due to ethnicity and geography. Different populations vary in genetics, dietary habits, and environmental factors such as air pollution or stress. Following bariatric procedures, patients’ diets and lifestyles influence microbial composition. A fiber-rich, polyphenol-rich diet may promote beneficial GM species stability, while a high-processed diet slows GM recovery.

A reduction in the F/B ratio often observed post-surgery is observed consistently in different studies and is correlated with weight loss. Beneficial species such as Bacteroidetes are involved in bile acid metabolism. Studies report the enhancement of FXR/TGR5 signaling. The gut barrier is stabilized by *Akkermansia* spp.

### 3.9. Persistence of Microbiota Changes Post-Bariatric Surgery

GM composition persists long-term after bariatric surgery; however, GM stability may differ due to individual factors such as dietary habits, weight maintenance, and baseline host metabolism. A partial reversion of GM shifts towards a pre-surgery profile may be due to weight regain and repeating dietary patterns. Consistently supplying a fiber-rich diet plays a significant role [62,63]. Weight regain increases the F/B ratio and lowers the diversity of GM. There is notably strong evidence for dietary modification having a beneficial effect on maintaining post-surgical microbiota and preventing metabolic decline.

### 3.10. Limitations and Future Directions

This literature review consists of several limitations. The selected articles were limited to original research published in English. Additionally, the review included articles from three different databases, which may have resulted in the omission of relevant studies available from other sources or in other languages. Furthermore, since the review focused primarily on human studies, direct experimental validation of gut microbiota mechanisms remains challenging.

GM is a relatively recent area of research, resulting in the limitation of long-term studies. As a result, this leaves uncertainties, including whether the clinical significance of GM shifts changes over time. Additionally, some available studies reported no significant changes in GM post-surgery.

Further research should focus on the long-term effects of GM shifts on mortality of patients after bariatric surgery, together with the regulation of probiotic supplementation after bariatric surgery. There is a potential area of further research regarding potential probiotics, prebiotics, or postbiotics for enhancing post-surgery outcomes.

## 4. Conclusions

Gut microbiota is a complex and independent ecosystem playing a central role in immunological, nutritional, and metabolism mediated functions. Under physiological conditions, it supports a host’s resistance to pathogens by activating immune cells and triggering an immune response. Dysbiosis, present in patients with obesity, is partially responsible for obesity, and the post-operative redistribution of gut microbiota in patients after bariatric surgery is still poorly understood. Patients with obesity show reduced microbial richness, compositional and functional changes, and low-grade inflammation, leading to increased body weight, increased fat mass, and DM2. The microbiota of patients with obesity is considered to have an increased capacity for energy harvesting. The SCFAs produced by oligosaccharides, fibers, and undigested proteins influence satiety hormone production, lipid metabolism, inflammation, and insulin sensitivity.

The impact of bariatric surgery on GM shifts differs depending on the type of procedure performed. RYGB induces the most significant changes, likely due to intestinal rearrangements and pH alterations. Bariatric surgery affects gut permeability by altering claudin, ZO-1, and occludin expressions, and TMAO levels. Further studies are needed to assess the clinical relevance of GM changes.

## Figures and Tables

**Table 1:** Changes in GM composition following different bariatric procedures.

Bariatric Procedure	Increased Bacterial Populations	Decreased Bacterial Populations	Additional Notes
**Roux-en-Y Gastric Bypass (RYGB)**	*Veillonella* spp., *Acidaminococcus* spp., *Slackia* spp., *Granulicatella* spp., *Akkermansia* spp., *Escherichia* spp., *Klebsiella* spp., Gammaproteobacteria	*Gemella* spp., *Clostridioides difficile*, *Clostridium hiranonis*, Lachnospiraceae, Ruminococcaceae	Increased microbial metabolites (TMAO); potential gut permeability changes; reduced LPS levels post-surgery.
**Sleeve Gastrectomy (SG)**	Bacteroidetes, Actinobacteria, *Blautia* spp., *Akkermansia* spp., *Eubacterium* spp., *Lactobacillus* spp., Cyanobacteria, *Haemophilus* spp.	Firmicutes, *Desulfovibrio* spp.	Lowered F/B ^1^ ratio; increased anti-inflammatory species; enhanced ketone body metabolism.
**Mini-Gastric Bypass (MGB)**	Limited research available	Limited research available	Similar weight reduction outcomes to RYGB; improved glycemic control and lipid profile.

Note: ^1^ Firmicutes/Bacteroidetes ratio.

## Data Availability

The data presented in this study are available on request from the corresponding author.

## Abbreviations

The following abbreviations are used in this manuscript:

GM	Gut Microbiota
SCFAs	Short-Chain Fatty Acids
WHO	World Health Organization
BMI	Body Mass Index
LPSs	Lipopolysaccharides
FFAR3	Free Fatty Acid Receptor 3
PYY	Peptide YY
DM2	Diabetes Mellitus Type 2
TLR4	Toll-Like Receptor 4
F/B	Firmicutes/Bacteroidetes (ratio)
IBS-D	Diarrhea Variant of Irritable Bowel Syndrome
IL-6	Interleukin 6
TNF-α	Tumor Necrosis Factor Alpha
INF-1	Interferon Type I
FMT	Fecal Microbiota Transplant
CDI	Clostridioides Difficile Infection
RYGB	Roux-en-Y Gastric Bypass
SG	Sleeve Gastrectomy
LGB	Laparoscopic Gastric Banding
TJ	Tight Junction
TMAO	Trimethylamine-N-Oxide
IL-1β	Interleukin 1 Beta
CVD	Cardiovascular Disease
ASCVD	Atherosclerotic Cardiovascular Disease
MGB	Mini-Gastric Bypass
SAGB	Single-Anastomosis Gastric Bypass
OAGB	One-Anastomosis Gastric Bypass
IFSO	International Federation for the Surgery of Obesity and Metabolic Disorders
